# Mitoregulin, a tiny protein at the crossroads of mitochondrial functioning, stress, and disease

**DOI:** 10.3389/fcell.2025.1545359

**Published:** 2025-03-05

**Authors:** Petr Sergiev, Olga Averina, Julia Golubeva, Mikhail Vyssokikh, Olga Dontsova

**Affiliations:** ^1^ Belozersky Institute of Physico-Chemical Biology, Lomonosov Moscow State University, Moscow, Russia; ^2^ Center for Life Sciences, Skolkovo Institute of Science and Technology, Skolkovo, Moscow Region, Russia; ^3^ Department of Chemistry, Lomonosov Moscow State University, Moscow, Russia; ^4^ Research Center for Obstetrics, Gynecology and Perinatology, Moscow, Russia; ^5^ Shemyakin-Ovchinnikov Institute of Bioorganic Chemistry, Russian Academy of Sciences, Moscow, Russia

**Keywords:** Mtln, mitochondria, small peptide, respiration, membrane, cardiolipin

## Abstract

Mitoregulin (Mtln) is a small mitochondrial protein that was only recently identified. Despite this, a substantial number of studies on its function have already been published. Although sometimes contradictory, these studies have revealed the localization of Mtln, its protein and lipid partners, and its role in lipid homeostasis, energy metabolism, oxidative stress, and other aspects of mitochondrial functioning. Moreover, research using knockout and transgenic mouse models has revealed the important role of Mtln in mammalian physiology. Metabolic changes, along with muscle, kidney, and fat-related phenotypes, have been linked to Mtln dysfunction. In this review, we summarize a comprehensive set of published data on Mtln. While controversies remain, we seek to offer a unified view of its functions, spanning molecular mechanisms to organism-level effects.

## 1 Introduction

Mitochondria, the powerhouses of the cell, play a central role in responses to several types of stress, including oxidative stress, ischemia–reperfusion, apoptosis, and senescence. Mitochondrial dysfunction may also be a cause of cellular stress. Mitochondrial function is precisely controlled by a set of approximately 1,100 proteins (Palmfeldt and Bross, 2017), among which a surprisingly high proportion are short peptides (sPEPs) (Calvo et al., 2016; Rath et al., 2021) encoded by short open reading frames. These sPEPs were almost overlooked by the standard genome annotation pipeline (Sergiev and Rubtsova, 2021). Over recent years, the development of ribosome profiling and improvements in mass spectrometry and the computational analysis of genome sequences has resulted in a burst of sPEP discoveries (Chugunova et al., 2018).

One recently identified sPEP is a product of a gene originally annotated as *LINC00116* in humans and *1500011k16Rik* in mice (Makarewich et al., 2018; Stein et al., 2018; Chugunova et al., 2019). When our group first investigated this gene, it had largely escaped the attention of the scientific community. However, by 2018–2019, several laboratories almost simultaneously published findings on the functional role of this small protein, now named “mitoregulin” (Mtln—also known as MOXI or MPM), which appeared to be an important part of the mitochondrial proteome (Makarewich et al., 2018; Stein et al., 2018; Chugunova et al., 2019; Lin et al., 2019; Lai et al., 2020). While many conclusions from studies of this protein agree, controversies remain, obscuring a precise understanding of the molecular mechanism that underlies Mtln function. In this review, we seek to summarize what is known about this small mitochondrial protein.

## 2 Mtln localization

**FIGURE 1:**
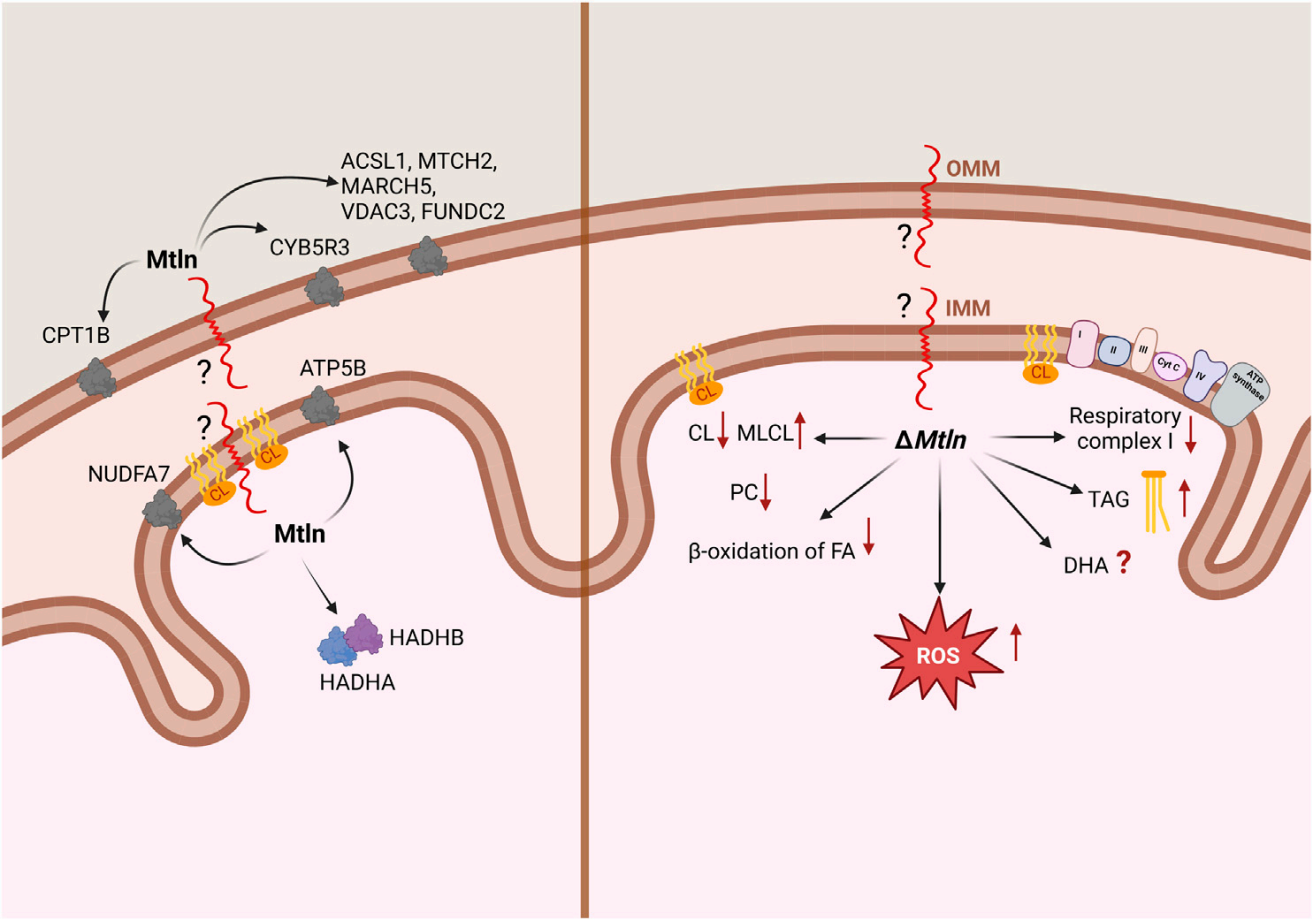
Summary of the data relating to Mtln localization and interaction **(A)** and the *Mtln*-knockout (KO) phenotype **(B)** at the level of mitochondria. IMM: inner mitochondrial membrane; OMM: outer mitochondrial membrane. Arrows in the left panel point to Mtln interaction partners; arrows in the right panel correspond to the phenotypes observed following *Mtln* inactivation. CL: cardiolipin; MLCL: monolysocardiolipin; PC: phosphatidylcholine; TAG: triacylglycerol; DHA: docosahexaenoic acid.

The initial understanding that the *LINC00116* gene codes for a protein, at least by our group (Chugunova et al., 2019), arose from conservation analysis. The most conserved region of the gene encodes an open reading frame of 56 amino acids. Further analysis by ours and other groups revealed that this sPEP is conserved among vertebrates, whereas invertebrates are generally believed to lack this gene (Makarewich et al., 2018; Stein et al., 2018; Chugunova et al., 2019). An assessment of nucleotide substitutions in the Mtln coding region revealed that most of them lead to the same or similar amino acids. A conserved hydrophobic region in the N-terminus of Mtln is likely to be a transmembrane segment, while the positively charged C-terminus is exposed for functional interactions outside the membrane. There is consensus among studies that Mtln is bound to the mitochondrial membrane, but discrepancies exist regarding whether it localizes to the inner or outer membrane (Figure 1A). Initial work by Makarewich et al. (2018) and Stein et al. (2018) and later by Lin et al. (2019) concluded that Mtln resides in the inner mitochondrial membrane (IMM) based on its resistance to proteinase K digestion. Recently, however, this conclusion has been challenged by the elegant study of Zhang et al. (2023). Their approach involved using a range of digitonin concentrations to selectively solubilize the outer mitochondrial membrane (OMM) and, more conclusively, a split-GFP system, in which a small fragment of GFP (GFP11) was tethered to the N- or C-terminus of Mtln, while the rest of the GFP molecule (GFP1-10) was artificially placed at several mitochondrial locations. Thus, the fluorescent GFP molecule could be formed only if both fragments, GFP11 and GFP1-10, appear in the same compartment. As a result, the authors demonstrated that Mtln resides in the OMM while its N-terminus faces the intermembrane space.

## 3 Partners of Mtln

The most valuable insight into the localization and function of Mtln is likely to be obtained from the identification of its binding partners. However, experiments employing affinity-tagged Mtln co-precipitation resulted in a list of protein interactors that only partially overlap across studies. For instance, Makarewich et al. (2018) found that Mtln interacts with the inner membrane proteins HADHA and HADHB, which are responsible for the β-oxidation of long-chain fatty acids, while Stein et al. (2018) reported that Mtln interacts with cardiolipin (CL), a highly specific lipid of the IMM. Our group (Chugunova et al., 2019) identified the outer membrane protein NADH-cytochrome b5 reductase 3 (CYB5R3) as a key Mtln-interacting partner. The list was later expanded to include ATP5B (Friesen et al., 2020), NUDFA7 (Xiao et al., 2022), *N*-acetyltransferase 14 (Nat14), and c-Jun (Li et al., 2023). More recently, Zhang et al. (2023) identified more partners of Mtln, including a set of OMM-localized proteins, such as CPT1B, ACSL1, MTCH2, MARCH5, VDAX3, FUNDC2, and CYB5B (Figure 1A). Additionally, Mtln has been reported to self-oligomerize (Linzer et al., 2024). There is little consensus among the results of different studies, with some exceptions. For example, two laboratories (Makarewich et al., 2018; Friesen et al., 2020) observed that Mtln interacts with HADHB, while both our group (Chugunova et al., 2019) and Zhang et al. (2023) noted that it interacts with CYB5R3. Mtln interacting partners are localized in the mitochondrial matrix, inner (IMM) and outer (OMM) mitochondrial membranes. Meanwhile, Mtln was initially reported to interact with respiratory chain supercomplexes (Stein et al., 2018) within the IMM and influence their activity. However, this assumption was subsequently disputed (Zhang et al., 2023). Mtln knockout appeared to increase the susceptibility of mitochondrial membranes to freezing (Stein et al., 2024), and thus freezing of mitochondria devoid of Mtln resulted in apparent dissociation of respiratory chain supercomplexes.

## 4 Respiratory phenotypes associated with Mtln deficiency

The main function of mitochondria is respiration. Four respiratory chain complexes transfer electrons from organic substrates to oxygen. Pyruvate generated via glycolysis is transported to mitochondria and converted to acetyl-CoA (AcCoA), concomitant with the production of NADH. AcCoA is oxidized during the tricarboxylic acid cycle, also yielding NADH molecules. The oxidation of fatty and amino acids also fuels the tricarboxylic acid cycle by providing AcCoA and other metabolites. Additionally, the NADH produced in the cytoplasm, such as through glycolysis, is transferred to mitochondria via malate and glutamate shuttle transporters. Ultimately, all mitochondrial NADH molecules are oxidized by respiratory complex I (CI), generating reduced ubiquinone QH_2_. Simultaneously, during the tricarboxylic acid cycle, succinate is oxidized to fumarate via respiratory complex II (CII). Apart from the production of AcCoA, fatty acid oxidation also results in the reduction of the ETF protein, which is later oxidized by ETF dehydrogenase. This process also generates reduced ubiquinone QH_2_. QH_2_ from all sources oxidized by respiratory chain complex III (CIII). Thus, several substrates can be used in model systems aimed at assessing the efficiency of respiratory complexes. For instance, the activity of NADH dehydrogenase in purified respiratory CI can be measured using NADH; pyruvate/malate or glutamate/malate are model substrates for the activity of respiratory chain CI in permeabilized cells or purified mitochondria; palmitoyl carnitine can be used to address the efficiency of both CI and fatty acid β-oxidation; finally, succinate is an exclusive substrate of respiratory chain CII.

The influence of Mtln on respiration has been studied by most groups which have investigated Mtln function using both cell lines and animal models. However, the results have varied to some extent. Our group revealed that Mtln inactivation in both cell (Chugunova et al., 2019) and mouse (Averina et al., 2023c) models affects respiratory CI activity irrespective of the substrate used (e.g., palmitoyl carnitine, pyruvate/malate, or glutamate/malate in permeabilized cells or purified mitochondria—Figure 1B). The activity of CI outside the context of the membrane (purified by immunoprecipitation) was found to be independent of Mtln (Chugunova et al., 2019). This suggested that Mtln is neither a *bona fide* component of CI nor is it involved in its assembly. It also does not favor the idea that Mtln is necessary for a particular type of CI fueling. Furthermore, respiration mediated by CII via succinate oxidation was found to be unaffected by Mtln inactivation, indicating that only CI, and not the rest of the respiratory chain, is dependent on it (Chugunova et al., 2019; Averina et al., 2023b, 2023c).

Two groups have reported that Mtln is involved in the β-oxidation of fatty acids. Makarewich et al. (2018) demonstrated that respiration efficiency is decreased in muscle and heart mitochondria from Mtln-KO mice when palmitoyl carnitine is used as the metabolic substrate, but not when pyruvate is used. They also showed that etomoxir, a specific inhibitor of carnitine palmitoyltransferase I (CPT-1), attenuates the difference in mitochondrial respiration between wild-type and Mtln-KO mice. Perfusion of the respiration substrates labeled with a stable ^13^C isotope followed by NMR analysis demonstrated that Mtln-KO mice preferentially oxidize carbohydrates at the expense of fatty acids (Makarewich et al., 2018). Friesen et al. (2020) also demonstrated that respiration efficiency was decreased in Mtln-KO adipocytes when palmitoyl carnitine served as the respiration substrate but was increased when glucose was utilized instead.

Lin et al. (2019) reported that respiration was dependent on Mtln when pyruvate is used as the respiratory substrate, as demonstrated by the XF Cell Mito Stress Test (Seahorse Bioscience). This implies that Mtln promotes CI-dependent respiration supported by substrates other than palmitoyl carnitine. However, in a later study, the same group found that respiration efficiency was increased with Mtln knockdown. The negative influence of Mtln on CI activity was attributed to its inhibitory interaction with the CI subunit NDUFA7 (Xiao et al., 2022). The discrepancy between the stimulatory (Lin et al., 2019) and the inhibitory (Xiao et al., 2022) effects of Mtln on mitochondrial respiration might be explained by differences in the cell lines used in these studies. The former experiments were conducted with murine myoblasts, while the latter were performed with human hepatocellular carcinoma cells. Further adding to the debate, Zhang et al. (2023) found no evidence of a dependence of respiration on Mtln.

Respiration in mitochondria occurs within the unique spatial architecture of cristae. Proper structural context is crucial for the activities of the respiratory complexes. Initially, Mtln was found to stimulate CI association into respiratory chain supercomplexes (Stein et al., 2018), although this finding was later challenged (Zhang et al., 2023). Mtln influence on supercomplex assembly appeared to be indirect and observed only if samples of mitochondria were frozen before the analysis by blue-native gel electrophoresis (Stein et al., 2024). Still, this finding is valuable since it indicates that a lack of Mtln makes inner mitochondrial membrane sensitive to freezing. Our group observed that the loss of Mtln decreased the amount of mitochondrial creatine kinase octamers (mtCK_8_) with the increase of the corresponding dimer (mtCK_2_) concentration (Averina et al., 2023b). This effect could also be explained by an impairment in the structural organization of the IMM upon Mtln depletion since cardiolipin located in the IMM is known to interact with mtCK and stabilize its octameric form.

## 5 The effect of Mtln on lipid composition

While debate regarding Mtln function persists, there is broad consensus that Mtln affects cellular and, presumably, mitochondrial lipid composition, thereby potentially influencing other mitochondrial functions. We undertook an analysis of lipid composition in Mtln-KO and corresponding control cell lines NIH 3T3 and NS0 (Chugunova et al., 2019). The major findings were a decrease in the concentrations of phosphatidylcholine lipids and an increase in triacylglycerol (TAG) levels in Mtln-KO cells (Figure 1B), with the biggest increase being detected in the concentrations of TAGs containing docosahexaenoic acid (DHA). Similarly, a subsequent study using C2C12 myoblasts deficient for Mtln reported an increase in total polyunsaturated fatty acids, with DHA concentrations exhibiting the most pronounced rise (Zhang et al., 2023). These findings contrast with Stein et al. (2024), who reported that the concentrations of DHA-containing triglycerides were decreased in the hearts of Mtln-KO mice whereas those of long-chain triglycerides were increased. These findings are puzzling, and the effect of Mtln deficiency on DHA-containing lipids remains contradictory.

The IMM contains a specific lipid, CL, which is needed to facilitate the bending of the IMM for cristae formation. CL is also known to interact directly with the respiratory chain complexes, especially CI, whose activity is critically dependent on this interaction (Jussupow et al., 2019). A direct interaction between CL and CI was demonstrated using an *in vitro* model system (Figure 1; Stein et al., 2018). Subsequent studies reported the CL levels being substantially reduced in muscles (Averina et al., 2023b) and kidneys (Averina et al., 2023c) of Mtln-KO mice, accompanied by a significant rise in the levels of monolysocardiolipin (MLCL), an intermediate product of CL damage and remodeling (Figure 1B). This finding was confirmed in a later study (Stein et al., 2024). CL is known to be damaged primarily by reactive oxygen species (ROS) (Dudek, 2017), and Mtln inactivation has been associated with an increase in ROS production in some (Stein et al., 2018; Choi and Kang, 2023), although not all (Chugunova et al., 2019), studies. Additionally, Mtln overexpression was observed to decrease ROS production (Choi and Kang, 2023; Figure 1B). The repair of oxidized CL proceeds via iPLA2γ-dependent fatty acid (FA) removal, yielding Mtln , whose concentrations are elevated in Mtln-KO mice. Cyb5r3, a partner of Mtln (Chugunova et al., 2019), plays a role in reducing ROS generation (Martin-Montalvo et al., 2016), and its levels were also found to be reduced in mice lacking Mtln (Averina et al., 2023c). Two major models for Mtln-mediated CL protection can be postulated, one involving direct interaction and the other involving indirect effects via Mtln protein partners, such as Cyb5r3.

## 6 The effect of Mtln on metabolite concentrations

**FIGURE 2:**
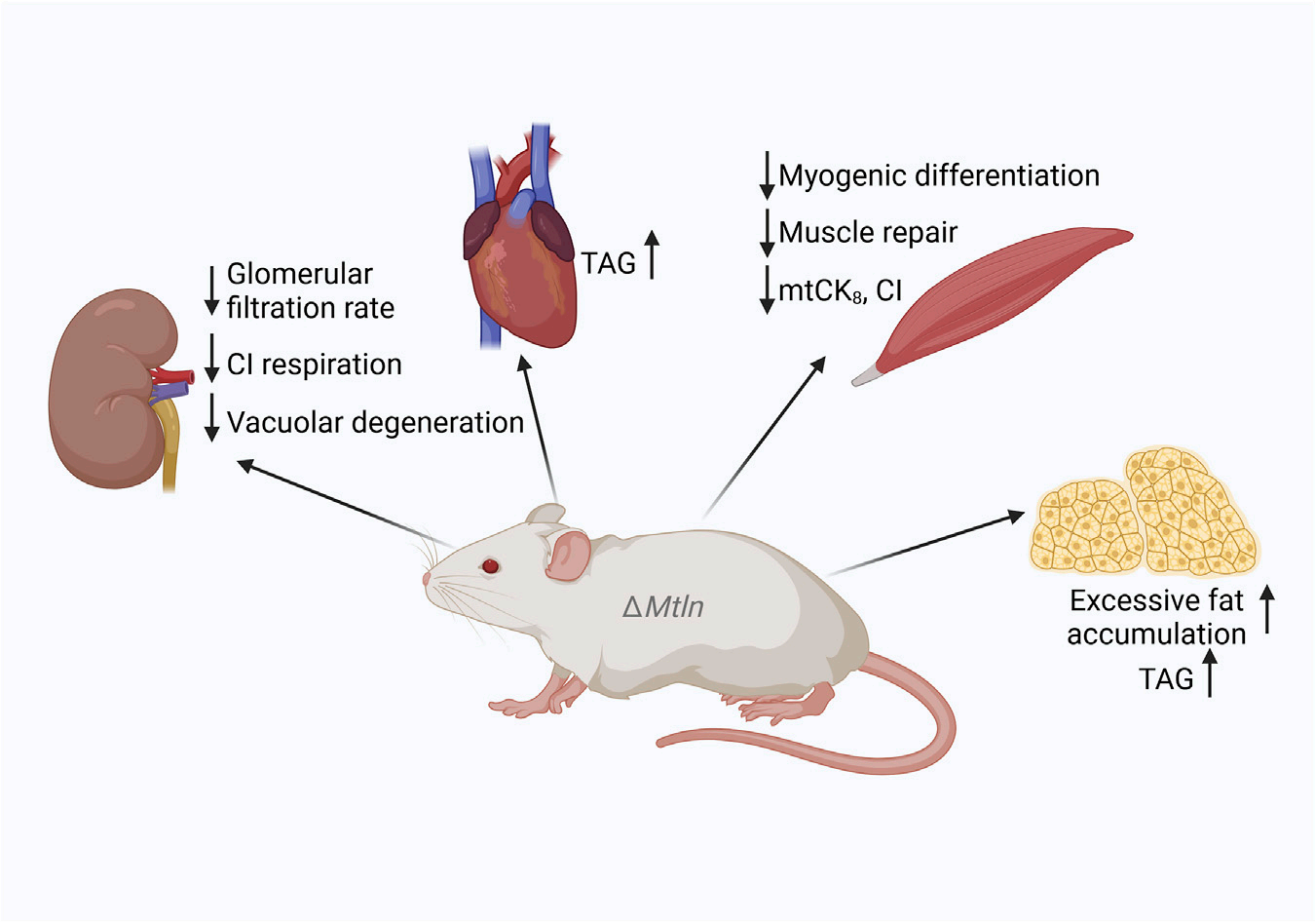
Summary of the phenotypes of *Mtln*-knockout (KO) mice. Downward arrows correspond to a decrease and upward arrows to an increase in function or activity following *Mtln* inactivation. CI: respiratory complex I; TAG: triacylglycerol.

To investigate the role of Mtln in mouse health and wellbeing, many Mtln-KO mouse models have been generated (Makarewich et al., 2018; Stein et al., 2018; Lin et al., 2019; Friesen et al., 2020; Wang et al., 2020; Averina et al., 2023a; Li et al., 2023; Zhang et al., 2023). Major characteristics, such as weight dynamics, have been addressed in several studies, resulting in somewhat controversial outcomes. Both neutral (Makarewich et al., 2018; Lin et al., 2019; Friesen et al., 2020; Averina et al., 2023a; Zhang et al., 2023) and inhibitory (Wang et al., 2020) effects on weight dynamics have been reported for Mtln-KO mice when fed a standard chow. Similarly, contradictory results regarding weight were obtained for Mtln-KO mice fed a high-fat diet, including an increase (Averina et al., 2023a), no change (Friesen et al., 2020), and a decrease (Zhang et al., 2023) in weight gain and fat accumulation. Serum triglyceride levels in Mtln-KO mice are also controversial, with both increases (Averina et al., 2023a) and decreases (Friesen et al., 2020) being reported. Nevertheless, increases in triglyceride accumulation have been observed in the hearts (Stein et al., 2024) and adipocytes (Friesen et al., 2020) of Mtln-KO mice, NS0 myeloma cells, and NIH 3T3 fibroblasts (Chugunova et al., 2019; Figures 1, 2). Curiously, an SNP associated with intramuscular fat accumulation was detected near the pig Mtln gene (Shen et al., 2024). Thus, despite controversies, it is likely that Mtln inactivation leads to fat accumulation, at least in some tissues or under some conditions.

The concentrations of a large set of serum metabolites were compared between Mtln-KO mice and wild-type controls (Averina et al., 2023a). An increase in lactate concentrations was observed following Mtln inactivation, which was expected given that lactate serves as a common marker for many human mitochondrial diseases as well as for mouse models of mitochondrial dysfunction (Averina et al., 2024). However, no other studies have observed an increase in serum lactate levels with Mtln deficiency (Makarewich et al., 2018; Zhang et al., 2023). Serum concentrations of several other metabolites were compared between the wild type and Mtln-KO mice. While we observed a decrease in the serum concentrations of citrate, malate, succinate, betaine, and glycine, concomitant with an increase in the contents of lysine and valine (Averina et al., 2023a), these results were not corroborated by Zhang et al. (2023).

In one study (Averina et al., 2023a), we also measured the serum concentrations of free fatty acids and acylcarnitines in Mtln-KO mice and found very moderate differences relative to wild-type controls. This was unexpected given that the major impact of Mtln inactivation is a deficiency in fatty acid β-oxidation (Makarewich et al., 2018; Stein et al., 2018; Friesen et al., 2020). Defects in fatty acid β-oxidation caused by HADHA dysfunction are expected to result in manyfold or even an order of magnitude difference in the levels of the acylcarnitines C14OH, C16OH, and C18:1OH (Sander et al., 2005)—not observed in Mtln-KO mice. Concurrently, we noted a decrease in the concentrations of the saturated free fatty acids C16:0, C18:0, and C20:0 and an increase in that of DHA. The latter was in agreement with our earlier observation in cell models, where we detected an increase in docosahexaenoic acid-containing triglyceride concentrations (Chugunova et al., 2019), and with that Zhang et al. (2023) relating to C2C12 myoblasts. However, this was not in line with an earlier study (Stein et al., 2024) which reported that the concentrations of DHA-containing triglycerides were decreased in the hearts of Mtln-KO mice.

It seems likely that Mtln inactivation influences the metabolic profile of mice to varying degrees, both positively and negatively. This influence also depends on subtle differences in genetic background as well as diet, age, sex, or other factors that are difficult to standardize across laboratories. At worst, the observed differences may simply result from random fluctuations.

## 7 The effect of Mtln on muscle physiology

The Mtln gene is highly expressed in muscles (Makarewich et al., 2018; Stein et al., 2018; Lin et al., 2019; Averina et al., 2023b)—expected, given that muscles, especially oxidative ones, contain a substantial number of mitochondria. Both forelimb grip strength (Lin et al., 2019; Averina et al., 2023b) and performance in the exhaustion running test were found to be decreased in mice devoid of Mtln (Wang et al., 2020). Mtln supports myogenic differentiation in C2C12 myoblasts (Lin et al., 2019; Wang et al., 2020) and muscle repair in several models (Lin et al., 2019; Wang et al., 2020; Averina et al., 2023b). A general decrease in muscle fiber diameter and a specific decrease in the diameter of muscle fibers with centralized nuclei at the regeneration stage following cardiotoxin-induced damage was observed (Lin et al., 2019). A reduction in the steady-state muscle fiber diameter in Mtln-KO mice was also noted (Wang et al., 2020). The same group reported that the weight of the tibialis anterior muscle was decreased in Mtln-KO mice, accompanied by a reduction in tetanic force. Muscle regeneration was found to be delayed following cardiotoxin-induced damage in mice lacking Mtln (Wang et al., 2020), in line with earlier findings (Lin et al., 2019). In our studies, we did not detect any differences in myofiber diameter due to Mtln inactivation. However, we did observe a tendency toward an increase in the number of myofibers with centrally located nuclear chains, indicative of augmented muscle remodeling (Averina et al., 2023b).

Muscle degeneration has also been reported in transgenic mice overexpressing Mtln (Makarewich et al., 2018; Figure 2). Acute Mtln inactivation in mouse hearts was achieved using AAV9 encoding Mtln-targeted guide RNAs in combination with cardiomyocyte-specific *Cas9* expression (Stein et al., 2024). This tissue-specific Mtln inactivation was found to increase the susceptibility of mice to myocardial infarction following heart ischemia reperfusion.

Although Gomori trichrome staining, which provides relatively low resolution, did not show evidence of mitochondrial damage in the muscles of Mtln-KO mice (Averina et al., 2023b), Makarewich et al. (2018) observed severe mitochondrial disorganization, including disrupted and enlarged cristae, using confocal microscopy.

Muscles use the creatine shuttle mechanism to transfer energy from the mitochondrial reticulum to myosin motors. As the diffusion of ATP is slower than that of creatine phosphate (CrP), a set of creatine kinases convert ATP to CrP and back, enabling CrP to act as a diffusible energy shuttle between the sites of ATP generation in mitochondria and ATP consumption by myosin. MtCK is a protein localized in the mitochondrial intermembrane space where it can persist in either a highly active octameric form mtCK_8_ or less active dimer mtCK_2_. The octameric form of the mitochondrial creatine kinase is bound to ATP/ADP antiporter and voltage-dependent anion channel (VDAC) (Vial, 2006; Puurand et al., 2018). Octamer formation is facilitated by an interaction with CL-containing IMM (Schlattner and Wallimann, 2000). We noted that the efficiency of muscle mitochondria respiration coupled with Cr phosphorylation was decreased in Mtln-KO mice (Averina et al., 2023b), which was paralleled by a decrease in mtCK octamerization and excessive CL damage (Figure 2). Thus, the creatine shuttle, an important mechanism in muscle physiology, was also found to be affected by Mtln inactivation. Combined, these observations indicate that suboptimal respiration and attenuated creatine shuttling may underlie the reduction in muscle performance detected following Mtln inactivation.

## 8 The effect of Mtln on kidney physiology

The kidney is the organ responsible for blood filtering and urine excretion. It is needed for the maintenance of water, ion, and solute balance, as well as for the excretion of metabolic waste and toxins. Kidney-related illnesses, such as chronic kidney disease, lead to kidney fibrosis and life-threatening conditions that require transplantation or constant dialysis. The kidney is enriched in mitochondria (Li et al., 2020), which are required to produce energy for its solute pumps. Many mitochondrial diseases, such as mitochondrial encephalomyopathy, lactic acidosis, and stroke-like symptoms (MELAS), myoclonic epilepsy with ragged-red fibers (MERRF), Pearson, Kearns–Sayre, and Leigh syndromes, result in kidney damage (O’Toole, 2014).

Several studies have focused on the role of Mtln in kidney physiology (Averina et al., 2023c; Li et al., 2023). In our studies (Averina et al., 2023a, 2023b), we found that the kidney exhibits relatively high Mtln expression, as determined by immunoblotting. However, this result was not replicated (Makarewich et al., 2018).

Mtln was observed to be highly expressed in patients with kidney fibrosis as well as in mouse models of kidney fibrosis, such as folic acid nephropathy and unilateral ureteral obstruction (Li et al., 2023). However, the upregulation of Mtln expression might not be a cause but rather a consequence of kidney damage, possibly a sort of a compensatory mechanism. Li et al. (2023) utilized artificial Mtln downregulation to investigate whether Mtln contributes to kidney fibrosis. The grade of kidney fibrosis induced by both folic acid nephropathy and unilateral ureteral obstruction was assessed by α-smooth muscle actin staining. The severity of kidney fibrosis was found to be attenuated in Mtln-KO mice and mice treated with locked nucleic acid antisense oligonucleotides that target Mtln-encoding mRNA (Li et al., 2023). The authors proposed that Mtln promotes a fibrotic response by acting as a bridge for the transcription factor c-Jun and the putative acetyltransferase Nat14. It would be surprising if a gene such as Mtln would be preserved in evolution if it was responsible for a pathology. More likely, Mtln is beneficial in conditions other than folic acid nephropathy and unilateral ureteral obstruction.

Our group investigated the age-dependent kidney-related physiological function of Mtln after unexpectedly detecting a severe kidney pathology in an 18-month-old Mtln-KO male mouse (Averina et al., 2023c). Follow-up studies on 12-month-old male mice revealed vacuolar degeneration in kidney proximal tubules in seven out of nine (78%) Mtln-KO mice compared with the four out of 14 (29%) recorded for wild-type mice. Simultaneously, we noted that glomerular filtration rates were reduced in 24-month-old female mice lacking Mtln (Averina et al., 2023c). Importantly, these pathologies were not detected in young, 6-month-old Mtln-KO mice, both male and female mice.

We also found that CI-dependent respiration was reduced while CL damage was enhanced in both the kidney (Averina et al., 2023c; Figure 2) and muscle mitochondria (Averina et al., 2023b) of Mtln-KO mice.

## 9 The effect of Mtln on human health

Despite being a very short gene with limited potential for genetic variability, Mtln was identified as being linked with serum triglyceridemia in genome-wide association studies (Surendran et al., 2012; Weissglas-Volkov et al., 2013). This finding aligns well with the increase in serum triglyceride levels and obesity observed in Mtln-KO model mice (Averina et al., 2023a).

Although sparse, several clinical studies have reported Mtln-related findings. Mtln was shown to be highly expressed in M2 (activated) macrophages at the sites of echinococcosis lesions (Ma et al., 2024). Mtln expression levels were also associated with later stages of echinococcosis infection in a mouse model, which ultimately resulted in liver fibrosis. Elevated Mtln expression has also been linked to kidney fibrosis, as mentioned above (Li et al., 2023). Another study described an increase in Mtln expression in osteoarthritis (Choi et al., 2024). Mtln upregulation was also observed in a synovial cell model following the activation of the inflammatory response by TNF-α and lipopolysaccharide. Similarly, Mtln KO in a synovial cell line resulted in the upregulation of inflammatory cytokine production (Choi et al., 2024).

Some studies have linked Mtln with cancer. For instance, Mtln expression is increased in lung cancer (Tomida et al., 2009). The inactivation of the Mtln gene in breast cancer cell lines was found to reduce the frequency of mitochondrial contacts with the ER under conditions of ER stress. In addition, the lack of Mtln resulted in the decreased viability of a breast cancer cell line following treatment with ER-stress inducers, such as tunicamycin (Choi and Kang, 2023). The loss of Mtln in lung cancer cell lines resulted in p53 activation and increased susceptibility to apoptosis, although these effects were attributed to the activity of Mtln-encoding RNA (Iwai et al., 2023). Proliferation and mobility in cervical cancer cell lines were also found to be dependent on Mtln expression (Lai et al., 2020). In contrast, hepatocellular carcinoma cell lines exhibited lower average Mtln expression, with elevated expression levels being indicative of a better prognosis (Xiao et al., 2022). In line with this, Mtln was shown to reduce metastatic potential, whereas its knockdown promoted migration and metastasis.

## 10 The extramitochondrial functions of Mtln

While the consensus view is that Mtln functions within mitochondria, alternative roles for Mtln have also been proposed. Most unusual, perhaps, is the finding that Mtln-encoding RNA functions as a *bona fide* lincRNA independently of its coding potential. As an RNA entity, LINC00116 binds to the PCBP2 protein, thereby enhancing its ability to repress p53 translation (Iwai et al., 2023). Alternatively, LINC00116 mRNA has been reported to enhance tumorigenesis by acting as a sponge for miR-106a, which, in turn, represses *c-Jun* expression (Lai et al., 2020). Notably, Mtln was observed to bind to and form a complex with both c-Jun and Nat14 in the nucleus (Li et al., 2023), which was assumed to activate fibrotic gene promoters.

## 11 Toward an integrative model of Mtln function

**FIGURE 3:**
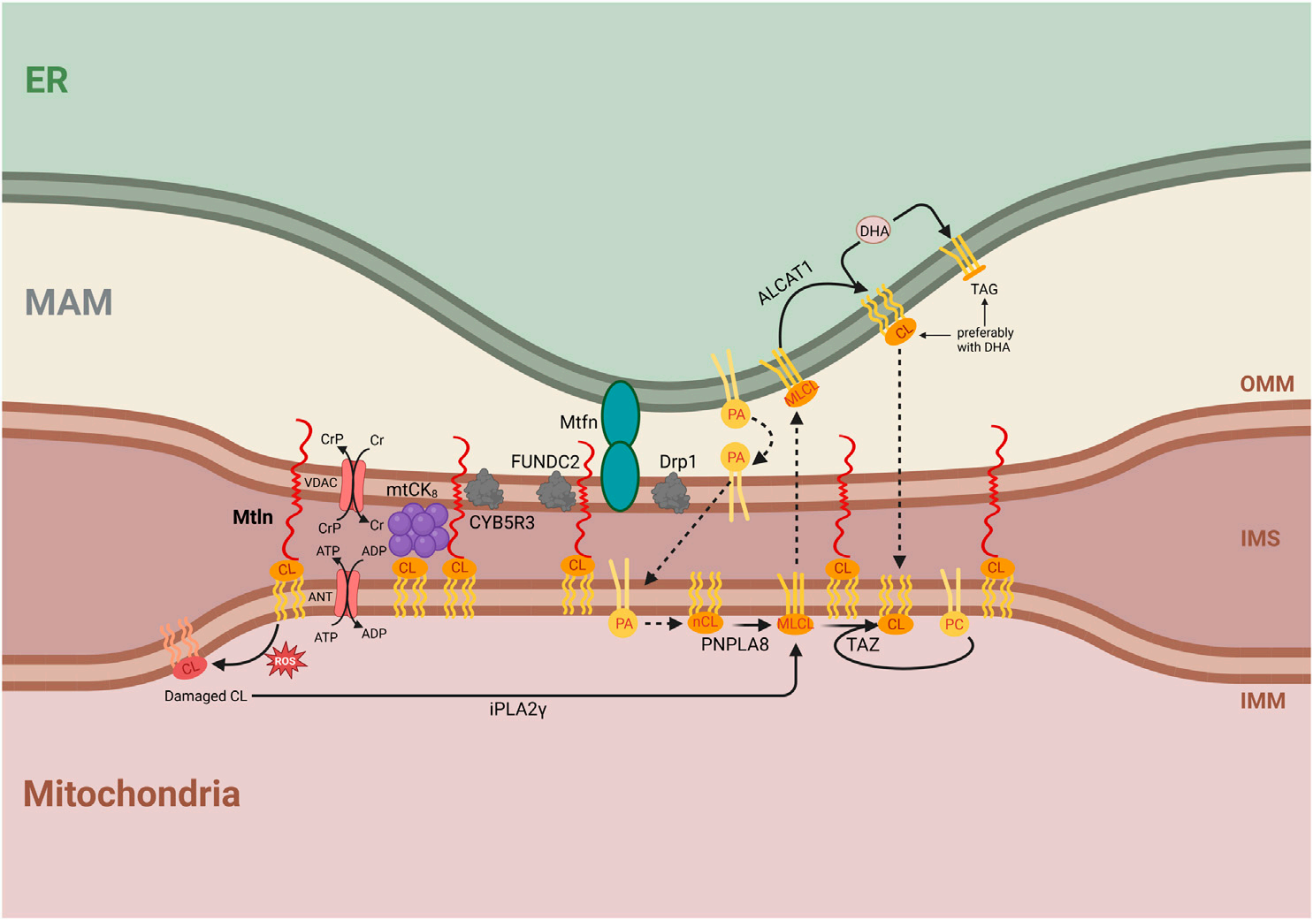
Integrative model for the molecular function of Mtln. Central is a proposed function of Mtln as a molecular tether between the inner mitochondrial membrane (IMM), outer mitochondrial membrane (OMM), and mitochondria-associated membrane (MAM). The labeled compartments are mitochondria, the intermembrane space (IMS), and the endoplasmic reticulum (ER). Shown are the interactions of Mtln with CL that arrange the contacts between the OMM and IMM. Shown are protein partners of Mtln, such as the CYB5R3, FUNDC2, mitofusin (Mtfn), and Drp1 localized in the membrane contact sites. The interaction of Mtln with CL could also protect CL from oxidation damage (shown on the left), which initiates CL repair via fatty acid removal by iPLA2γ forming monolysocardiolipin (shown below). CL synthesis is initiated by phosphatidic acid transport at the site of contacts between MAM, OMM, and IMM membranes (shown in the center). Initially formed newly synthesized cardiolipin (nCL) is processed by PNPLA8 to monolysocardiolipin (MLCL). MLCL might be transported to the ER membrane, where it is repaired by ALCAT1 with the addition of DHA. Alternatively, taffazin (TAZ) transfers a fatty acid, preferentially C18:2, from phosphatidylcholine (PC) to MLCL inside the mitochondria. CL synthesis, damage, and repair pathways are shown with an emphasis on the essential influence of the intermembrane exchange processes, which require membrane tethering. The Cr shuttle system is shown as dependent on the interaction with CL and facilitated by the contacts between the membranes. Mitochondrial creatine kinase (mtCK) is shown as an octamer, stabilized by an interaction with both IMM and OMM. ATP/ADP antiporter and VDAC used to arrange Cr shuttling are shown next to mtCK_8_. Mtln interaction with CL is shown to stabilize intermembrane contacts made by the mtCK_8_.

Initially, the Mtln-encoding gene was misannotated as a long non-coding RNA with no known protein product. Since its identification, there has been a surge in studies related to the localization, partners, molecular function, and physiological role of this protein. While every reported finding about Mtln has been met with some degree of controversy, several of its features have obtained support from at least two independent research groups. Accordingly, a clearer picture of the function of Mtln is beginning to emerge, as summarized below in Figure 3.

Despite conflicting reports (Makarewich et al., 2018; Stein et al., 2018), a recent robust study demonstrated that Mtln is a mitochondrial peptide, the transmembrane segment of which is inserted into the OMM, while its N-terminus faces the intermembrane space (Zhang et al., 2023). This arrangement is consistent with its reported ability to interact with CL (Stein et al., 2018) within the IMM. Moreover, taken together, these results suggest that Mtln functions as a link between the IMM and the OMM (Figure 3).

The pleiotropic and sometimes contradictory phenotypes observed with Mtln inactivation in both cell culture and mice support Mtln KO manifestations being highly influenced by subtle differences in the genotypes of mice or the nature of cell cultures, the amounts of specific nutrients, such as fatty acids, and the age of mice. These findings make it unlikely that Mtln is a *bona fide* component of a specific enzymatic protein complex, or a much stronger and robust phenotype would be anticipated. The location of Mtln in the OMM while its N-terminus is facing the IMM and interacts with the IMM lipid CL supports it playing a structural role by acting as a membrane tether (Figure 3). The possibility that Mtln acts in the tethering of the IMM and OMM may explain its effect on mitochondrial creatine kinase octamerization (Averina et al., 2023b). The octameric form of mtCK is known to interact with the cardiolipin located in the IMM and tether both IMM and OMM (Rojo et al., 1991). Additional IMM to OMM bridging by Mtln should stabilize mtCK_8_ binding to both mitochondrial membranes (Schlattner and Wallimann, 2000; Schlattner et al., 2006), thus stabilizing mtCK_8_ formation.

The transport of lipids between the IMM and the OMM is linked to the transport between the OMM and endoplasmic reticulum (ER) membranes (mitochondria-associated membranes [MAMs]) as many lipid components are exchanged between ER and IMM via OMM (see below). Mtln was reported to be present in the OMM–MAM contact proteome, as revealed by Contact-ID, a method for specifically labelling and purifying proteins localized at the contact sites between particular cellular compartments (Kwak et al., 2020). The contact proteome was similarly enriched in Mtln-interacting partners, such as Cyb5r3 (Chugunova et al., 2019; Zhang et al., 2023) and FUNDC2 (Zhang et al., 2023). The proteins Drp1 and Mfn2, which are responsible for mitochondrial fission (Smirnova et al., 2001; Tábara et al., 2024) and fusion (Santel and Fuller, 2001; Tábara et al., 2024), respectively, are localized to OMM–MAM contact sites (de Brito and Scorrano, 2008; Friedman et al., 2011). Mtln inactivation decreases the abundance of Drp1 (Choi and Kang, 2023) while increasing that of Mfn2 (Averina et al., 2023c). In addition, Mtln inactivation was shown to reduce the number of OMM–MAM contacts, especially under ER stress (Choi and Kang, 2023). This suggests that Mtln may not only contribute to the tethering between OMM and IMM but also to the stabilization of OMM–MAM contacts via its C-terminal domain and protein partners (Figure 3). Hypothetically, Mtln might facilitate the formation of the triple IMM–OMM–MAM contacts.

Contact sites between the IMM and OMM, as well as between the OMM and MAM, are important for lipid exchange, which is necessary for CL synthesis. The CL precursor, phosphatidic acid (PA), is imported into the IMM from the MAM through membrane contact sites (Jiang et al., 2022). This suggests that a reduction in the number and stability of contact sites between the MAM and OMM and the OMM and IMM would affect PA import into the mitochondria and consequently impede CL synthesis. Newly synthesized CL must be converted to its mature form by fatty acid exchange, initiated by iPLA2γ-dependent fatty acid removal, which is also used for the repair of CL following ROS-induced damage. CL remodeling may proceed via mitochondrial TAZ protein, which transfers a fatty acid, preferentially linoleic acid (C18:2), from phosphatidylcholine to MLCL (Schlame et al., 2017). Alternatively, MLCL could be exported to the ER. In the latter case, ALCAT1 converts MLCL into CL, with a preference for DHA incorporation (Figure 3). Several studies have reported differences in DHA contents with Mtln depletion. We observed that DHA-containing triglycerides accumulate in *MTLN*-KO cell lines whereas the phosphatidylcholine content is depleted (Chugunova et al., 2019). This might be explained by a deficiency in MLCL traffic between the mitochondria and ER, such that an excess of DHA would be used for triglyceride synthesis, while phosphatidylcholine would be depleted due to excessive TAZ-mediated MLCL remodeling. Another consequence of Mtln inactivation—the accumulation of MLCL and the reduction of CL contents (Averina et al., 2023b; Stein et al., 2024)—might also be explained by reduced CL remodeling associated with transmembrane transport (Figure 3). Other explanations are possible, such as the protection of CL from oxidative damage through direct interaction with Mtln or the prevention of an increase in ROS production mediated by Mtln. The loss of Mtln was found to be associated with enhanced ROS generation (Stein et al., 2018), while a partner of Mtln, Cyb5r3 (Chugunova et al., 2019; Zhang et al., 2023), which is known to protect cells against oxidative damage (Hall et al., 2022), seems to decay under conditions of Mtln deficiency (Averina et al., 2023c).

## 12 Future perspectives

Despite a surge in studies on Mtln, there is still no unequivocal mechanistic understanding of Mtln function. To address this knowledge gap, membrane tethering by Mtln can be assessed in *in vitro* experiments, while the effects of Mtln on lipid transportation across mitochondrial membranes require a careful examination of lipidome composition in the IMM, OMM, and MAM after Mtln inactivation. Given recent outstanding progress in mitochondrial structural biology, it may soon be possible to directly observe Mtln.
